# The Role of TSC1 in the Macrophages Against *Vibrio vulnificus* Infection

**DOI:** 10.3389/fcimb.2020.596609

**Published:** 2021-01-27

**Authors:** Xian-Hui Huang, Yao Ma, Han Lou, Na Chen, Ting Zhang, Liu-Ying Wu, Yi-Ju Chen, Meng-Meng Zheng, Yong-Liang Lou, Dan-Li Xie

**Affiliations:** ^1^ Department of Microbiology and Immunology, School of Laboratory Medicine and Life Science, Wenzhou Medical University, Wenzhou, China; ^2^ Key Laboratory of Laboratory Medicine, Ministry of Education of China, Wenzhou, China; ^3^ Department of Infection and Immunity, Wenzhou Key Laboratory of Sanitary Microbiology, Wenzhou, China; ^4^ Department of Laboratory Medicine, Dong Yang People’s Hospital, Jinhua, China; ^5^ Department of Pathology, School of Basic Medical Science, Wenzhou Medical University, Wenzhou, China; ^6^ Department of Laboratory Medicine, Jinshan Hospital of Fudan University, Shanghai, China

**Keywords:** *Vibrio vulnificus*, tuberous sclerosis complex 1, mammalian target of rapamycin, macrophage, polarization, bactericidal activity

## Abstract

*Vibrio vulnificus* (*V. vulnificus*) is an estuarine bacterium that is capable of causing rapidly fatal infection in humans. Proper polarization and bactericidal activity of macrophages play essential roles in defending against invading pathogens. How macrophages limit *V. vulnificus* infection remains not well understood. Here we report that tuberous sclerosis complex 1 (TSC1) is crucial for the regulation of *V. vulnificus*-induced macrophage polarization, bacterial clearance, and cell death. Mice with myeloid-specific deletion of TSC1 exhibit a significant reduction of survival time after *V. vulnificus* infection. *V. vulnificus* infection induces both M1 and M2 polarization. However, TSC1 deficient macrophages show enhanced M1 response to *V. vulnificus* infection. Interestedly, the absence of TSC1 in myeloid cells results in impaired bacterial clearance both *in vivo* and *in vitro* after *V. vulnificus* infection. Inhibition of the mammalian target of rapamycin (mTOR) activity significantly reverses *V. vulnificus*-induced hypersensitive M1 response and resistant bactericidal activity both in wild-type and TSC1-deficient macrophages. Moreover, *V. vulnificus* infection causes cell death of macrophages, possibly contributes to defective of bacterial clearance, which also exhibits in a mTORC1-dependent manner. These findings highlight an essential role for the TSC1-mTOR signaling in the regulation of innate immunity against *V. vulnificus* infection.

## Introduction

*Vibrio vulnificus* (*V. vulnificus*) is an emerging estuarine bacterium of coastal waters worldwide, such as the United States, Japan, China, South Korea, and Mexico (Zhao et al., 2015; Heng et al., 2017). The bacterium can cause severe gastrointestinal disease, wound infections, and highly fatal sepsis. The pathogenesis of virulence factors produced by *V. vulnificus* was well defined, however how the host immune response to this bacterium is still unclear. Macrophages are one of the most prominent cell lineages of innate immunity, which play an important role as sentinels against microbes (Ishii et al., 2008; Diacovich and Gorvel, 2010). The receptors on the macrophage cell surface (toll-like receptors, TLRs) or in the cytoplasm (nucleotide-binding oligomerization domain-like receptors, NLRs), recognize various bacterial components and infectious agents and induce macrophage activation (Ishii et al., 2008).

The different stages of macrophage activation are described as M1 (classical) and M2 (alternative) polarization. M1 and M2 macrophages are functionally polarized in response to bacterial infection (Mege et al., 2011). M1 macrophages are activated by LPS and IFN-γ to elaborate proinflammatory cytokine production and tissue inflammation to promote cell apoptosis (Mosser and Edwards, 2008). After LPS stimulation, M1 macrophages produce proinflammatory cytokines, such as TNF-α, IL-6, IL-12, and IL-1β (Mogensen, 2009; Patel et al., 2017). M2 macrophages are stimulated by Th2 cytokines IL-4 and IL-13 to promote helminthic immunity, fibrosis, allergy, and immunomodulation (Odegaard et al., 2007; Sica and Mantovani, 2012). Generally, macrophages are polarized toward an M1 response to kill the invading organisms and activate adaptive immunity in the early stage of bacterial infection (Liu et al., 2014). However, an excessive or prolonged M1 program is deleterious for the host after *Escherichia coli*, *Toxoplasma gondii*, or *Pseudomonas aeruginosa* infection (Benoit et al., 2008; Kong et al., 2015; Chen et al., 2018). Additionally, during sepsis, the accumulated M1-type cytokines in the circulatory system are highly correlated with mortality (Bozza et al., 2007). Rapamycin, an mammalian target of rapamycin complex 1 (mTORC1) inhibitor, was reported to inhibit M2 macrophage polarization (Jin et al., 2018).

We previously reported that *V. vulnificus*-induced the mTORC1 activation and server inflammatory responses in macrophages (Xie et al., 2017). The serine-threonine kinase mTOR is a critical regulator in both innate and adaptive immune cells. In mammal cells, mTOR exists in two complexes: mTORC1 and mTORC2, which control the specific effector functions in the innate immune cells, including metabolism, phagocytosis, cytokine production, and macrophage polarization (Covarrubias et al., 2015; Weichhart et al., 2015). Whereas the pathogenesis of *V. vulnificus*-induced inflammation *via* mTORC1 activation in macrophages remains mostly unknown. Tuberous sclerosis complex 1 (TSC1) and TSC2 are tumor suppressor genes. TSC1 and TSC2 negatively regulate mTORC1 activity. TSC1 associates with TSC2 to form a heterodimer. TSC1 is also essential for TSC2 activity by the maintenance of TSC2 protein stability (Pan et al., 2012; Xie et al., 2012). Genetic loss of either TSC1 or TSC2 leads to tumorigenesis correlated with overactivated mTORC1 signaling. Recently studies have shown that TSC1/2-mTORC1 signaling plays significant roles in M2 polarization responding to IL-4 (Byles et al., 2013). However, little is known regarding the role of TSC1 mediated macrophage polarization in the regulation of *V. vulnificus* infection.

In this study, we report that TSC1^f/f^-LyzCre^+^ mice show a significantly elevated death after *V. vulnificus* infection by comparing it with C57BL/6J wild-type mice. The *V. vulnificus*-infected TSC1^f/f^-LyzCre^+^ mice have a marked defect of bactericidal activity in the spleen, lung, and liver. In wild-type mice, *V. vulnificus* infection causes M1 polarization. We confirm that TSC1 deficient macrophages are skewed towards M1 phenotypes. Besides, mTORC1 overactivation in the TSC1 deficient macrophage also results in increased cell-death after *V. vulnificus* infection. However, rapamycin treatment, at least in part, blocks *V. vulnificus*-induced M1 polarization in both wild-type and TSC1 deficient macrophages. Our results indicate that TSC1 mediated mTORC1 activity plays a crucial role in the regulation of the macrophage viability, polarization, and bacterial clearance, which might contribute to host defense in *V. vulnificus* infection.

## Materials and Methods

### Mice

We purchased the 6- to 8-wk-old C57BL/6J mice from the Chinese Academy of Science Shanghai SLAC Laboratory Animal Center. The Tsc1 flox mice and *LysozymeCre* (LyzCre) transgenic mice were purchased from The Jackson Laboratory. Tsc1 flox mice were bred with *LysozymeCre* mice to induce TSC1 deletion in myeloid cells.

### Bacterial Strains and Cell Culture

L-929 cells were purchased from the Cell Bank of the Chinese Academy of Science in Shanghai and cultured in RPMI1640 containing 10% heat-inactivated fetal bovine serum (Tianhang Bio) and penicillin-streptomycin (50 IU/ml and 50 mg/ml, Beyotime). The China General Microbiological Culture Collection Center provided the *Vibrio vulnificus* CGMCC 1.1758 strain, which was grew at 37°C in brain heart infusion broth (BHI) or on the BHI blood agar plate. The procedures of *V. vulnificus* that were used in this study were followed from the standard biological hazards at Biosafety Level 2 and the safety procedures of Wenzhou Medical University Laboratory Safety Department.

### Reagents and Antibodies

Anti-mF4/80-APC, anti-mCD11b-PE, PE Annexin V Apoptosis Detection Kit I andCytofix/Cytoperm™ and perm/wash solutions were purchased from BD Biosciences Pharmingen (San Diego, CA, USA). Anti-miNOS-PE was purchased from eBioscience. Anti-mCD206-APC was purchased from BioLegend. Hamartin/TSC1 (D43E2) Rabbit mAb, phospho-S6 Ribosomal Protein (Ser235/236) (D57.2.2E) XP^®^ Rabbit mAb, phospho-p70 S6 Kinase (Thr389) (D5U1O) Rabbit mAb, and Cleaved Caspase-3 (Asp175) (5A1E) Rabbit mAb were purchased from Cell Signaling Technology (CST). Phosphatase Inhibitor Cocktail (Millipore) was purchased from Sigma-Aldrich. Mouse TNF-α Elisa kit, Mouse IL-6 Elisa kit, Mouse IL-1β Elisa kit, LEGENDplex™ Mouse Th1/Th2 Panel (8-plex) (San Diego, CA, USA). LIVE/DEAD Fixable Blue Dead Cell Stain Kit (for UV excitation) was purchased from Invitrogen.

### RNA Sequencing

J774A.1 cells with 6 h *V. vulnificus* infection or PBS treatment were used for RNA sequencing. Briefly, the cells were lysed in TRIzol and subjected to Annoroad Co. for RNA extraction, sequencing, and transcriptome profile analysis. The significantly differentially expressed genes were identified when we compared the normalized reads count between *V. vulnificus* and PBS groups with p < 0.05 and |Log2FoldChange| > 0.263. The significance of the gene ontology term enrichment was estimated using Fisher’s Exact Test (*p* value).

### *V. vulnificus* Infection of Bone Marrow-Derived Macrophages (*BMMϕs*) *In Vitro*

The bone marrow cells were isolated from femurs and tibiae and cultured them with a 10% L-929 cell culture medium, as described previously (Pan et al., 2012). Bone marrow cells were cultured with 1640 medium (Gibco) containing 10% (v/v) FBS and 15% (v/v) L929 conditional medium for seven days to obtain bone marrow-derived macrophages. A total of 1 × 10^6^ *BMMϕs* from both TSC1^flox/flox^ LysM-Cre (TSC1 KO) and C57BL/6J (WT) were equally seeded in six-well plates or 35 mm dishes and cultured at 37°C for overnight. We then added *V. vulnificus* at the indicated multiplicity of infection (2MOI) to the cells for 2 h and 4 h. The supernatant was collected at the indicated time for cytokine quantification. The infected cells were used for flow cytometry analysis, RT-qPCR analysis, and Western blot analysis.

### *In Vivo* Infection

We used i.p. injection to infect male C57BL/6J and TSC1^flox/flox^ LysM-Cre mice with *V. vulnificus*. For survival experiments, we injected mice with 0.5 × 10^8^ CFU of *V. vulnificus* suspended in 200 μl of PBS or PBS alone. The mice were observed every 6 h for 48 h, and the mice that survived for 48 h should have been humanely killed. We euthanized and exsanguinated the mice 4 h after infection for blood and tissue collection. The *V. vulnificus* engulfed by phagocytes consisting of organs could be counted by grinding the organs completely with PBS and detected with BHI blood agar.

### Quantitative PCR Analysis

The inflammatory response of macrophages was induced by 2MOI of *V. vulnificus* for 2 h and 4 h. Total RNA was extracted with TRIzol (Omega Bio-Tek). The reverse transcription was performed with PrimeScript Reverse Transcriptase (RR037A, Takara Bio) according to the manufacturer’s instructions. Real-time qPCR was performed as previously described (Xie et al., 2012). The primers were shown in **Supplementary Table 1**. To determine the relative induction of cytokine mRNA in response to various stimuli, the mRNA expression was normalized with β-actin and then was calculated using 2^-ΔΔCT^ method.

### Western Blot Analysis

Cell lysates preparation and Western blot analysis were performed by following the previous protocol (Xie et al., 2012). Briefly, Macrophages were cultured in RPMI 1640 medium with 10% FBS in six-well plates. Cells were treated with 2 MOI of *V. vulnificus* for 1 or 4 h. Cells were washed twice in cold PBS, then were lysed in RIPA buffer (Beyotime Bio) with protease and phosphatase inhibitor cocktails (Sigma) for 15 min at 4°C. Protein concentration was determined by Bradford assay. Protein samples were analyzed by SDS–PAGE gel and transferred to PVDF membrane. The membrane was blocked with 5% non-fat dried milk in TBST (100 mM Tris–HCl pH 7.5, 150 mM NaCl, 0.05% Tween20) for 1 h, then incubated with indicated primary antibodies overnight on a shaker at 4°C. The appropriate HRP-coupled secondary antibody was then added and was detected through ECL chemiluminescence (BioRad). β-actin was used as a protein loading control.

### Cytokine and Nitric Oxide Production Assay

Cytokine concentration was determined using ELISA kits for IL-1β, TNFα, and IL-6 (BioLegend). We purchased a nitric oxide Test Kit from Nanjing Jiancheng Bio to measure nitric oxide production in the supernatant. Briefly, the supernatant from cultured-cells with indicated treatment was collected and centrifuged at 4°C 2,000 g per 15 min, and then was analyzed by following the manufacturer’s instruction.

### Measurement of Cytokines by Multiplex Flow Assay

The plasma inflammatory cytokines were detected by flow cytometry with LEGENDplex™ Mouse Th1/Th2 Panel (8-plex, Biolegend) kit by following our previous protocol (Xie et al., 2017). Briefly, we assessed the cytokine concentrations in serum and supernatants of cultured cells according to the manufacturer’s instructions. We collected data using a BD FACSAria II and analyzed them with LEGENDplex data analysis software (Biolegend).

### Intracellular and Extracellular Bacterial Growth

Cells were infected with 2 MOI of *V. vulnificus* for 4 h and washed with PBS. Supernatant was diluted and plated them on blood BHI plates for 12 h. Then 100 μg/ml gentamicin was added to kill extracellular bacteria. After incubation at 37°C for 30 min, cells were washed twice with PBS and treated with 0.1% Triton-X100. The cell lysis was plated on blood BHI plates for 12 h at 37°C for the bacterial count.

### Flow Cytometry

Cells were infected with 2 MOI of *V. vulnificus* at 37°C for 4 h. For the M1/M2 discrimination flow cytometry experiment, cells were stained with CD206-APC and iNOS-PE after fixation and permeabilization by BD Biosciences Cytofix/Cytoperm™ and perm/wash solutions. For the cell apoptosis assay, dying cell was identified by using the BD PE Annexin V Apoptosis Detection Kit I according to the manufacturer’s protocol. Data were collected by using flow cytometry.

### Histological Analysis

After the spleen, liver, and lung were fixed in 4% paraformaldehyde, we imbedded the fixed organs in paraffin, then cut thin sections, and following standard procedures, stained them with hematoxylin and eosin.

### Statistical Analysis

Data were presented as mean ± SEM and analyzed for statistical differences using the Prism 6.01/GraphPad software. Statistical significance was analyzed using the Student *t*-test. *P*-values less than 0.05 were considered significant.

## Results

### Macrophage Viability and Activation Are Modulated by *V. vulnificus* Infection

**Figure 1:**
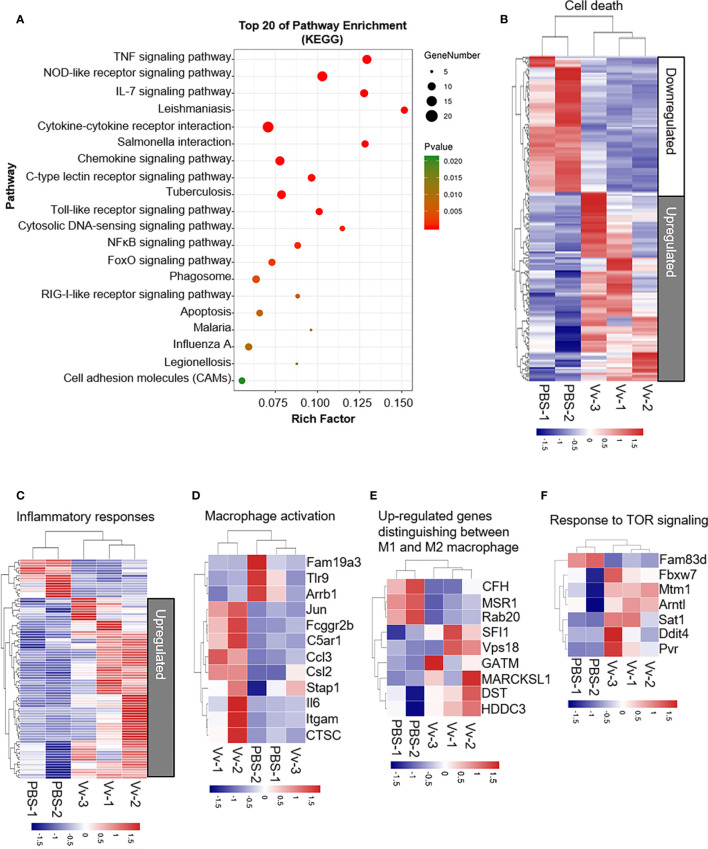
*V. vulnificus* induces multiple cellular biological events and pathways in macrophages. **(A)** KEGG pathway analysis of genes 6 h after two MOI of *V. vulnificus* infection. Genes are categorized into the most represented pathways in which the gene products are involved (P < 0.05). **(B–F)** Transcriptional profiles of J774A.1 cells left uninfected (PBS treatment) or infected with *V. vulnificus* (Vv) for 4 h (PBS: n = 2; Vv: n = 3). The expression of genes is presented as centered and scaled log_2_ fluorescence intensity (blue and red keys) grouped by product functions, including cell death **(B)**, inflammatory responses **(C)**, macrophage activation **(D)**, upregulated genes distinguishing between M1 and M2 macrophages **(E)**, and response to TOR signaling **(F)**.

Our first aim was to investigate host cellular function during the interaction of macrophages with *V. vulnificus*. To understand how *V. vulnificus* causes inflammation, cell death, and activation in macrophages, we performed RNA-Seq and transcriptomic analysis in J774A.1 cells before and after *V. vulnificus* infection. RNA-seq analysis revealed substantial changes in the macrophages transcriptome after 6 h of infection. The KEGG pathway enrichment analysis demonstrated that the top pathways involved in TNF signaling, NOD-like receptor signaling, IL-7 signaling, and cytokine-cytokine receptor signaling pathways were overrepresented in the upregulated genes after 6 h of infection (**Figure 1A**). Next, we analyzed the differentially expressed genes after 6 h of infection using the hallmark gene sets from the Molecular Signatures Database (MSigDB) and *GO Term* revealed a substantial upregulation of genes involved in the process of cell death, inflammatory response, macrophage activation, macrophage polarization, and TOR signaling (**Figures 1B–F**). These results indicated that *V. vulnificus* was likely to trigger macrophage inflammatory response, especially inducing M1 polarization, which is accompanied by activation of TOR signaling.

### Constitutive mTORC1 Activity by Myeloid-Specific TSC1 Deletion Impairs the Survival of Mice and Host Bactericidal Activity After *V. vulnificus* Infection

**Figure 2:**
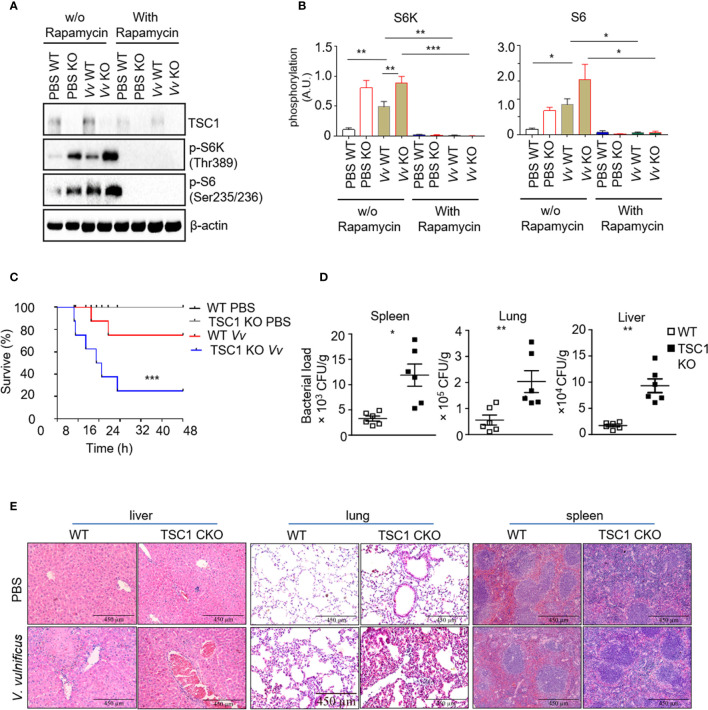
Mice with a myeloid-specific TSC1 deficiency are hypersensitive to *V. vulnificus* infection **(A)** Western blots determined the levels of TSC1, p-S6K, p-S6, and β-actin in *V. vulnificus*-infected WT and TSC1 KO macrophages. **(B)** Quantification of relative intensity of western blot bands. **(C)** Kaplan–Meier plots of WT and TSC1 CKO mice survival after *V. vulnificus* injection were shown. P values were determined using Log-rank tests. **(D)** Quantification of bacterial burden in the liver, lung, and spleen from *V. vulnificus*-infected WT and TSC1 CKO mice. **(E)** Histopathologic analysis (H&E) of liver, lung, and spleen tissue from *V. vulnificus*-infected and -uninfected wildtype and TSC1 CKO mice. Data shown are representative of at least three experiments. *P < 0.05; **P < 0.01; ***P < 0.001 was determined by Student *t*-test.

In the previous studies, we reported that *V. vulnificus*-infection could cause mTORC1 activation in the macrophage *in vivo* and *in vitro*. Whereas the consequence of *V. vulnificus*-induced mTORC1 activation in macrophages to the host remains unknown. We determined the mTOR signaling in the WT and TSC1 deficient *BMMϕs* with *V. vulnificus* infection. *V. vulnificus*-infected WT *BMMϕs* exhibited elevated S6K1 and S6 phosphorylation (**Figures 2A, B**). These results are consistent with our previous studies (Xie et al., 2017). While phosphorylation of S6K1 and S6 were also constitutive higher in both PBS-treated and *V. vulnificus-infected* TSC1 deficient *BMMϕs* (**Figures 2A, B**). Moreover, the phosphorylation of S6K1 and S6 in both WT and TSC1 deficient *BMMϕs* could be inhibited by rapamycin. In addition to this, we observed that the protein level of TSC1 was decreased in *V. vulnificus*-infected *BMMϕs* after rapamycin treatment, which could be possibly caused by the reduction of mTORC1-dependent protein synthesis. Because mTORC1 signal plays an important role in the regulation of numerous protein synthesis. We also observed that the protein level of TSC1 was deceased after rapamycin treatment, which possibly caused by the reduction of mTORC1-dependent protein synthesis. These results confirmed that *V. vulnificus* could induce mTOR activation in the murine macrophages. We also tested the role of TSC1-mTOR signaling in the myeloid cells *in vivo* during *V. vulnificus* infection. It was found that TSC1^f/f^-LyzCre^+^ mice had a shorter survival time than the WT mice (**Figure 2C**). Furthermore, TSC1^f/f^-LyzCre^+^ mice had a significantly higher bacterial burden in the liver, spleen, and lung compared to WT mice (**Figure 2D**). To understander the reason that causes the early death in *V. vulnificus*-infected TSC1 KO mice, we performed H&E staining for the liver, lung, and spleen from wildtype and TSC1 KO mice with or without *V. vulnificus* infection. In both *V. vulnificus*-infected wildtype and TSC1 CKO mice, liver damage was mainly located near vessels (**Figure 2E**). Moreover, *V. vulnificus*-infected TSC1 KO liver tissue revealed more severe focal inflammation and portal inflammation as compare with wildtype mice (**Figure 2E**). Similar with the pathology of liver, the lung and spleen from *V. vulnificus*-infected TSC1 KO mice also showed enhanced inflammatory cells infiltration (**Figure 2E**). Together, these results suggested that TSC1 in the macrophages contributed negatively to the susceptibility of mice to *V. vulnificus.* It possibly due to the enhanced-inflammatory damage in the organs, reduction of bactericidal activity, and dysfunction of macrophages.

### TSC1 Impairs M1 Polarization but Enhances M2 Polarization Induced by *V. vulnificus*

**Figure 3:**
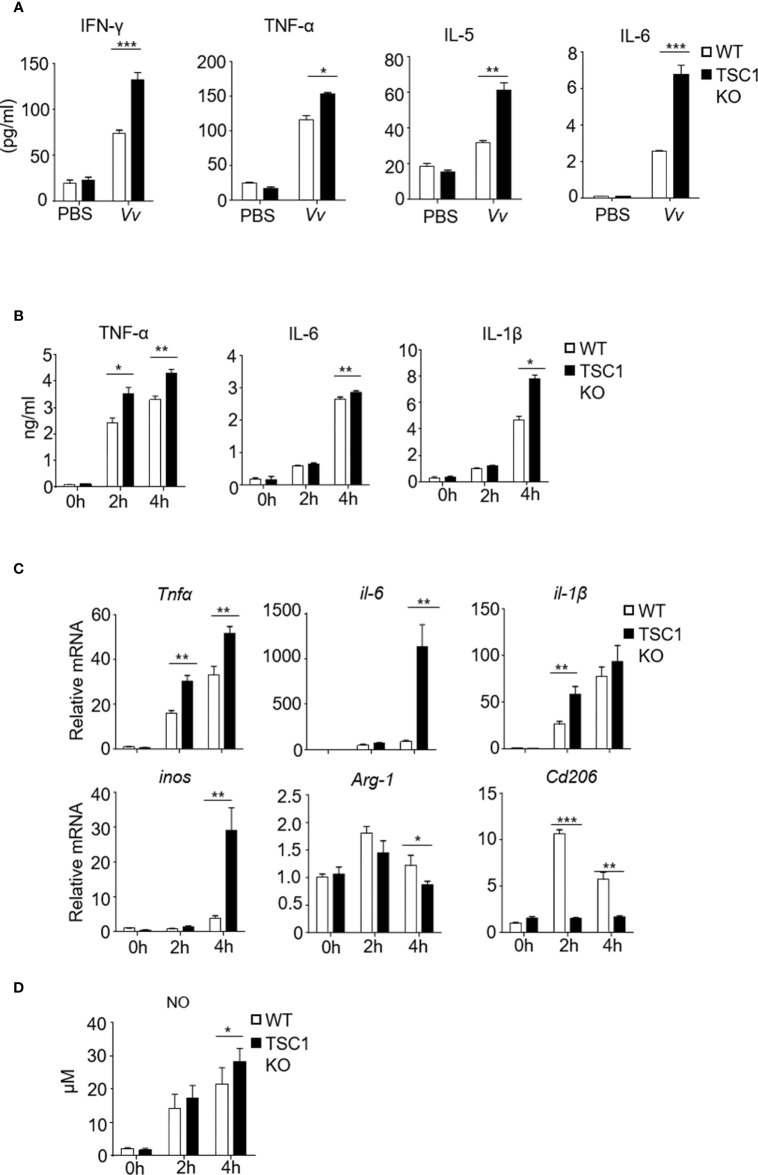
The absence of TSC1 in macrophages have defective of M2 phenotype and enhance M1 polarization by *V. vulnificus*. **(A)** Inflammatory cytokine production in the serum from WT and TSC1KO mice after *V. vulnificus* infection for 4 hours. **(B)** The production of TNF-α, IL-6, and IL-1β in the supernatant from TSC1 KO and WT *BMMϕs* after 2MOI of *V. vulnificus* infection for 2 and 4 h. **(C)** RT-PCR determined the mRNA expression of indicated-genes from WT and *BMMϕs* 4 h after *V. vulnificus* infection. **(D)** The bar figure shows the nitric oxide level of WT and *BMMϕs* after *V. vulnificus* infection. Data shown are representative of at least three experiments. *P < 0.05; **P < 0.01; ***P < 0.001 was determined by Student *t*-test.

The function of macrophages depends on proper activation and polarization into distinct subtypes with individual effector functions, such as the M1 and M2 macrophage subsets (Weichhart et al., 2015). M1 macrophages contribute to the clearance of invading organisms against bacterial infection (Benoit et al., 2008). The *V. vulnificus*-infected WT mice exhibited upregulation of IFN-γ, TNF-α, IL-6, and IL-5 production in the serum (**Figure 3A**). Notably, in contrast with the WT mice, the *V. vulnificus*-infected TSC1^f/f^-LyzCre^+^ mice produced enhanced IFN-γ, TNF-α, IL-6, and IL-5 production in the serum (**Figure 3A**). These results indicated that the absence of TSC1 in the myeloid cells might promote the Th1 and M1 response by *V. vulnificus*. *V. vulnificus* infection also caused elevated TNF-α, IL-6, and IL-1β in both mRNA and protein levels (**Figures 3B, C**). Moreover, macrophages with TSC1 deletion expressed a higher level of TNF-α, IL-6, and IL-1β compared with WT cells after *V. vulnificus* infection (**Figures 3B, C**). However, the mRNA transcription of M2 macrophage biomarker *Arg-1* and *Cd206* was significantly decreased in TSC1 KO *BMMϕs* (**Figure 3C**). Additionally, *V. vulnificus* infection significantly increased the NO production in the macrophages both in WT and TSC1 KO macrophages (**Figure 3D**). The change in NO production by *V. vulnificus* both in WT and TSC1 KO macrophages was correlated with similar changes in inducible nitric oxide synthase (iNOS) mRNA expression, suggesting that increased NO production by *V. vulnificus* was due to the expression of iNOS. Additionally, as shown in **Figure S1A** and **S1B**, *V. vulnificus* infection induced upregulation of the numbers of iNOS^+^ M1 macrophages and CD206^+^ M2 macrophages in WT *BMMϕs*. Whereas, in contrast to WT *BMMϕs*, TSC1 KO *BMMϕs* shown increased M1 macrophages but a reduction of M2 macrophages after *V. vulnificus* infection. Thus, these results indicated that TSC1 possibly drives *V. vulnificus*-induced M2 polarization and suppresses M1 polarization. Overall, these results demonstrated that TSC1 negatively regulated *V. vulnificus*-infected macrophages preferred polarization to M1 instead of M2.

### *V. vulnificus*-Induced M1 Polarization and Apoptosis in a mTORC1-Dependent Manner

**Figure 4:**
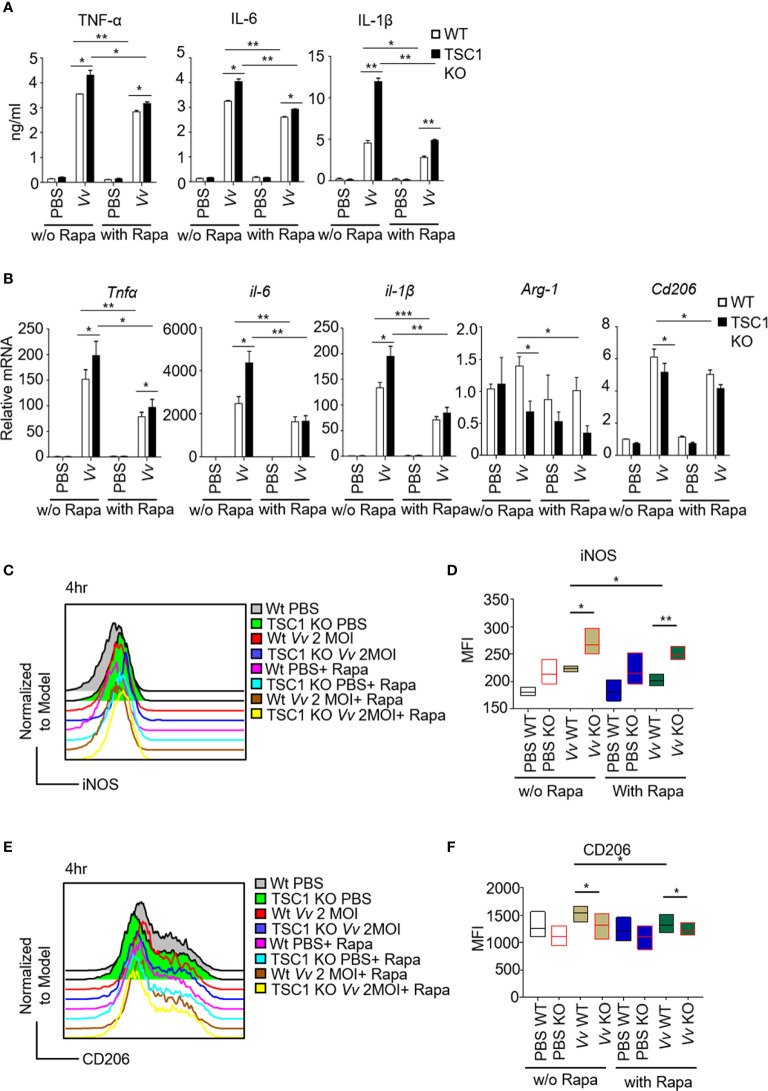
The M1 polarization in TSC1 KO macrophages by *V. vulnificus* are dependent on mTORC1 activity. **(A)** The production of TNF-α, IL-6, and IL-1β in *V. vulnificus*-infected (200 nM, 1-h pretreatment). **(B)** Expression of indicated genes in *V. vulnificus*-infected WT and TSC1 KO macrophages in the presence or absence of rapamycin pretreatment. PBS was used as control. **(C–F)** Representative histograms showing iNOS **(C)** and CD206 **(E)** expression gated from CD11b and F4/80 double-positive *BMMϕs* after *V. vulnificus* infection. Bar graphs show the MFI of iNOS **(D)** and CD206 **(F)** in the CD11b^+^F4/80^+^ live *BMMϕs* with indicated-treatments. Data shown are representative of at least three experiments. *P < 0.05; **P < 0.01; ***P < 0.001 was determined by Student *t*-test.

Recently studies have shown that TSC1 attenuated M1 polarization in an mTOR-independent manner fashion but increased M2 polarization *via* inhibition of mTORC1 activity (Byles et al., 2013; Zhu et al., 2014). To investigate whether TSC1-mTOR signaling is essential for *V. vulnificus*-induced M1 polarization, we infected the WT and TSC1 KO *BMMϕs* with two multiplicity of infection (MOI) of *V. vulnificus* pretreated with or without rapamycin. We observed that the upregulation of both protein and mRNA expression of TNF-α, IL-6, and IL-1β in the *V. vulnificus*-infected WT and TSC1 KO macrophages were significantly inhibited by rapamycin (**Figures 4A, B**). Moreover, *V. vulnificus*-induced mRNA levels of *Arg-1* and *Cd206* in both WT and TSC1 KO macrophages were also could be partially blocked by rapamycin treatment (**Figure 4B**). We further tested the iNOS and CD206 expression in *BMMϕs* after *V. vulnificus* infection. TSC1 KO *BMMϕs* increased iNOS expression and impaired the CD206 expression by comparing with WT *BMMϕs* after *V. vulnificus* infection (**Figures 4C–F**). However, the enhanced iNOS expression in *V. vulnificus*-infected TSC1 KO macrophages can be overcome by rapamycin treatment (**Figures 4C, D**). Thus, these results indicated that *V. vulnificus*-induced M1 and M2 polarization were both dependent on mTORC1 activity.

**Figure 5:**
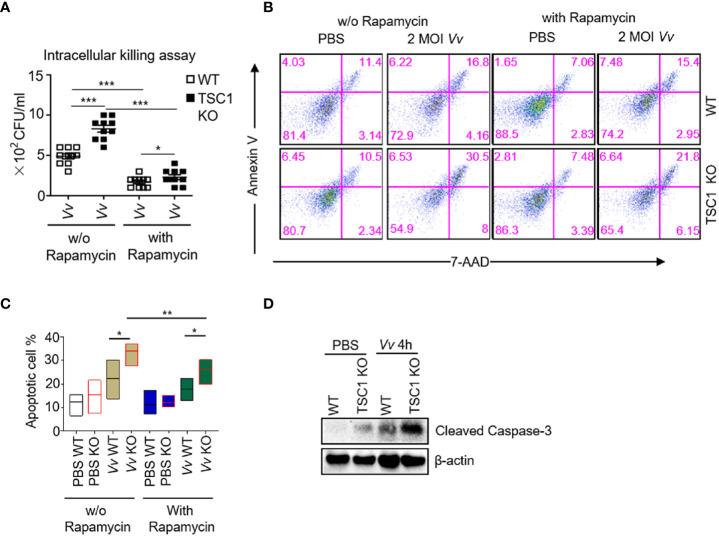
The apoptosis and bactericidal activity in TSC1 KO macrophages by *V. vulnificus* are dependent on mTORC1 activity. **(A)** Intracellular killing assay with viable *V. vulnificus* in WT and TSC1 KO macrophages with or without rapamycin pretreatment. **(B)** Representative dot plots showing Annexin V and 7AAD staining cells gated from CD11b and F4/80 double-positive *BMMϕs* after *V. vulnificus* infection. **(C)** Bar graphs show the frequency of Annexin V^+^7AAD^-^ plus Annexin V^+^ 7AAD^+^ gated from CD11b^+^F4/80^+^ *BMMϕs* with indicated-treatments. **(D)** Western blots determined the levels of cleaved-Caspase 3 in the *BMMϕs* with indicated-treatments. Data shown are representative of at least three experiments. *P < 0.05; **P < 0.01; ***P < 0.001 was determined by Student *t*-test.

Next, we further determined whether mTORC1 activity affected the bactericidal activity to clearance the invaded *V. vulnificus* in the macrophages. Interestingly, although the TSC1 KO *BMMϕs* showed enhanced M1 polarization by *V. vulnificus* infection (**Figure 4**), TSC1 KO macrophages exhibited more viable of intracellular *V. vulnificus* in the cells compared with WT cells (**Figure 5A**), which could be significantly blocked by abolishing the mTORC1 activity with rapamycin treatment. However, there was no significant difference in the extracellular bacterial burden from the supernatant between *V. vulnificus*-infected WT and TSC1 KO *BMMϕs* (**Figure S2**). These results suggested that the bactericidal activity to clearance the invaded *V. vulnificus* was negatively regulated by mTORC1 activity and the sustained activation of mTORC1 in TSC1 deficient macrophages possibly promote the intracellular bacterial survival. A previous study revealed that *V. vulnificus* could cause apoptosis in the macrophages (Kashimoto et al., 2003). We hypothesize that *V. vulnificus* triggered mTORC1 activation may positively regulate the *V. vulnificus*-induced apoptosis in the macrophages. To examine this, we analyzed the apoptosis of *V. vulnificus*-infected WT and TSC1 KO macrophages with or without rapamycin treatment. As shown in **Figures 5B**, **C**, the absence of TSC1 caused higher apoptosis in *V. vulnificus*-infected macrophages compared with WT cells. Rapamycin treatment could partially rescue the cell apoptosis from *V. vulnificus* in both WT and TSC1 KO macrophages (**Figures 5B, C**). Additionally, TSC1 KO macrophages increased the expression of cleaved-Caspase3 after *V. vulnificus* infection by comparing with WT macrophages (**Figure 5D**). This observation confirmed that constitutively active mTORC1 could induce Caspase 3 cleavage, which promote *V. vulnificus*-induced apoptosis in macrophages. It possibly impaired the bactericidal activity of macrophages.

## Discussion

*Vibrio vulnificus* is a Gram-negative bacterium that causes a rapidly progressive fatal septicemia and necrotizing wound infection resulting in extensive tissue damage (Chen et al., 2017). Mortality was up to 50% in septic patients, with most of them dying within 48 h with a fulminating course after infection (Chiang and Chuang, 2003; Horseman and Surani, 2011). Macrophages are one of the frontline cells of host defense against pathogenic microorganisms or pathogen invasion. Macrophages polarize to M1 response to kill the invading pathogens (such as *Salmonella typhi*, *Salmonella typhimurium*, and *Mycobacterium tuberculosis*, etc.) by the production of various proinflammatory cytokines including TNF-α, IL-6, and IL-1β in the early stage of bacterial infection (Sica and Mantovani, 2012; Liu et al., 2014). Subsequently, to neutralize excessive inflammatory response, macrophages polarized to M2 response to protect the host from excessive injury, which can be seen in the recovery period in patients with typhoid fever (Thompson et al., 2009).

Previous studies have shown that TSC1 can restrain M1 polarization response to LPS but enhanced M2 polarization response to IL-4 (Byles et al., 2013; Zhu et al., 2014). Converging the result that macrophages polarized to both M1 and M2 after *Vibrio vulnificus* infection, we hypothesize that TSC1 is critical for regulation of macrophage polarization by *Vibrio vulnificus*. In our study, we observed enhanced M1 macrophages with decreased M2 macrophages in TSC1 KO macrophages infected with *V. vulnificus*. To figure out whether and how TSC1-mediated macrophage polarization impacts the resistance of macrophages to *Vibrio vulnificus*. We analyzed the viability and bactericidal capacity of macrophages after *Vibrio vulnificus* infection. The results proved that TSC1KO macrophages exhibited lower viability and worse bactericidal capacity after *Vibrio vulnificus* infection compared to WT cells. These results support that TSC1 inhibits M1 polarization but promotes M2 polarization after *Vibrio vulnificus* infection and benefits for macrophages to kill the intracellular bacteria by suppression of the *V. vulnificus*-triggered cell-death. Previously, the relationship between macrophage polarization and *V. vulnificus* infection is unclear. In this study, we also found *V. vulnificus* infection could cause both M1 and M2 polarization in a mTORC1 dependent manner.

The current reports have shown that sustained activation of mTORC1 in TSC1KO macrophages attenuates IL-4 induced M2 polarization, but TSC1 controls M1 polarization independent of the mTOR pathway. To figure out whether TSC1 regulates *V. vulnificus*-infected macrophages polarization is dependent on the mTOR pathway. We used rapamycin in TSC1 KO macrophages before incubated with *V. vulnificus.* The results proved that TSC1 mostly inhibited M1 polarization and partially enhanced M2 polarization *via* inhibition of mTORC1 activity by *V. vulnificus*. We also found TSC1 was critical for the promotion of the bactericidal capacity of macrophages against *V. vulnificus*. Thus, TSC1 inhibits *V. vulnificus*-infected M1 polarization and contributes to terminate the *Vibrio vulnificus via* mTORC1 pathway. Previous studies revealed that *V. vulnificus* infection resulted in apoptosis and autophagy in the macrophages (Kashimoto et al., 2003; Song et al., 2016). NLRP3 and mTOR signaling also is required for phagolysosome formation in the macrophages after *V. vulnificus* infection (Huang et al., 2020). Our data suggested that overactivation of mTORC1 in the macrophages promoted the caspase 3 cleavage and apoptosis. However, the important issue of whether autophagy is involved in *V. vulnificus*-infected TSC1 KO macrophages is still needed to be investigated in the future.

NO is crucial for macrophages to kill the pathogens. Although *V. vulnificu*s is vulnerable to the exposure of NO produced by murine macrophage (Kim et al., 2019). We have observed elevated *inos* transcription and NO production in *V. vulnificus*-infected TSC1 KO *BMMϕs* by comparing with WT controls. However, the defects of TSC1 in the *BMMϕs* still could not restore the intracellular clearance of *V. vulnificus* and rescue the mice from *V. vulnificus* infection. Previous studies also revealed that *V. vulnificus* infection could induce macrophage apoptosis (Kashimoto et al., 2003). Thus, the impaired bactericidal activity partially due to *V. vulnificus* infection could cause more cell death in TSC1 deficient *BMMϕs.* Therefore, we have further tested the apoptosis of WT and TSC1 KO *BMMϕs* after *V. vulnificus* infection. *V. vulnificus* induces more apoptotic cells in TSC1 deficient *BMMϕs*, which could be partially blocked by rapamycin. Together, we believe that the enhanced apoptosis in *V. vulnificus*-infected TSC1 KO *BMMϕs* might contribute to impair iNOS and NO mediated bactericidal activity.

In *Salmonella typhimurium* infection, the M1 macrophages usually play as a safeguard to induce M1 polarization and control the infection (Benoit et al., 2008). M1 polarization also can prevent *Mycobacterium tuberculosis* escape from the phagosomes (Chacon-Salinas et al., 2005). Whereas, the excessive M1 program is also deleterious to the host (Mehta et al., 2004). In *Streptococcus pneumoniae*-infected mice, the mortality associates with lung inflammation and the presence of M1 macrophages (Smith et al., 2007). Our results showed that a higher level of bacterial burden in different organs and server inflammatory responses, which correlated with the increased mortality in the TSC1^flox/flox^ LysM-Cre^+^ mice upon *V. vulnificus* infection. Taken together, TSC1 KO mice exhibit more susceptibility of mice to *V. vulnificus*.

Overall, our results indicated that *V. vulnificus*-infected TSC1 KO macrophages lead to excessive M1 polarization, a higher level of cell death, and imperfect bactericidal activity, which might be resulted in more susceptibility of mice against *V. vulnificus*. Thus, the TSC1-mTORC1 signaling pathway negatively regulates the host against the *V. vulnificus* infection, which could be a potential target for the treatment of *V. vulnificus* infection.

## Data Availability Statement

The raw data supporting the conclusions of this article will be made available by the authors, without undue reservation.

## Ethics Statement

The animal study was reviewed and approved by the Wenzhou Medical University Animal Care and Use Committee.

## Author Contributions

D-LX, Y-LL, and M-MZ conceived and designed experiments. D-LX, Y-LL, MZ, X-HH, YM, HL, NC, TZ, L-YW, and Y-JC analyzed the data. D-LX, M-MZ, X-HH, YM, HL, NC, TZ, L-YW, and Y-JC conducted the experiments. D-LX and X-HH wrote the manuscript. All authors contributed to the article and approved the submitted version.

## Funding

This work was supported by the National Natural and Science Foundation of China (31400764 to D-LX, 81772229 to Y-LL), Provincial Natural and Science Foundation of Zhejiang (LY13H190007 to D-LX), Wenzhou Municipal Science and Technology Bureau (Y20150113 to D-LX).

## Conflict of Interest

The authors declare that the research was conducted in the absence of any commercial or financial relationships that could be construed as a potential conflict of interest.
