# Supplementary material for: The Role of TSC1 in the Macrophages Against Vibrio vulnificus Infection
Source: Front Cell Infect Microbiol. 2021 Jan 27;10:596609. doi: 10.3389/fcimb.2020.596609 (PMC7873526; doi:10.3389/fcimb.2020.596609)
Supplement: Supplementary file 1 [file DataSheet_1.docx]

Supplementary Material

## 1.Supplementary Figures


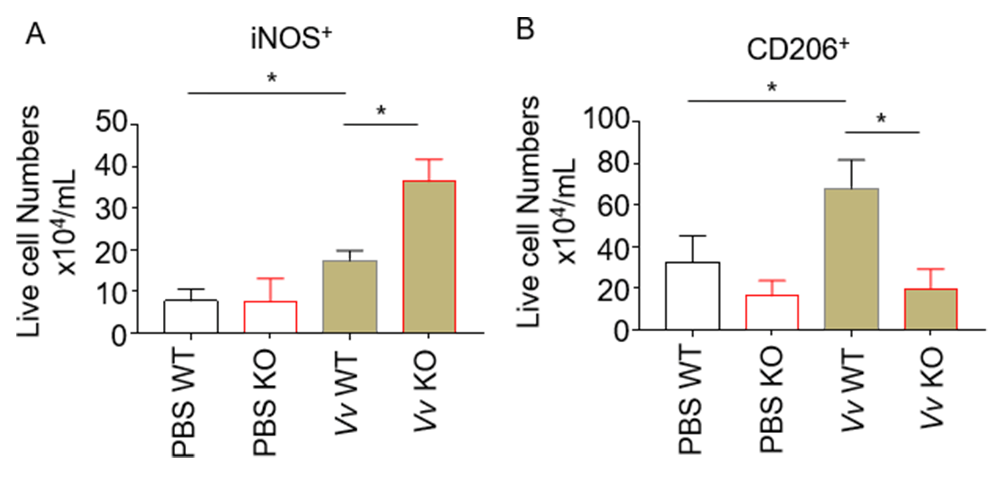


**Supplementary Figure 1.** **The M1 and M2 macrophages cell numbers in *V. vulnificus*-infected WT and TSC1 KO *BMMϕs*.** The frequency of iNOS^+^ M1 macrophages and CD206^+^ M2 macrophages were analyzed by flow cytometry. The iNOS^+^ M1 macrophages and CD206^+^ M2 macrophages in the indicated treatments were gated from live CD11b^+^F4/80^+^ *BMMϕs*. The absolute number of M1 macrophages (A) and M2 macrophages (B) were shown in the bar figures. Data shown are representative of at least three experiments. *, *P* < 0.05 was determined by Student *t*-test.


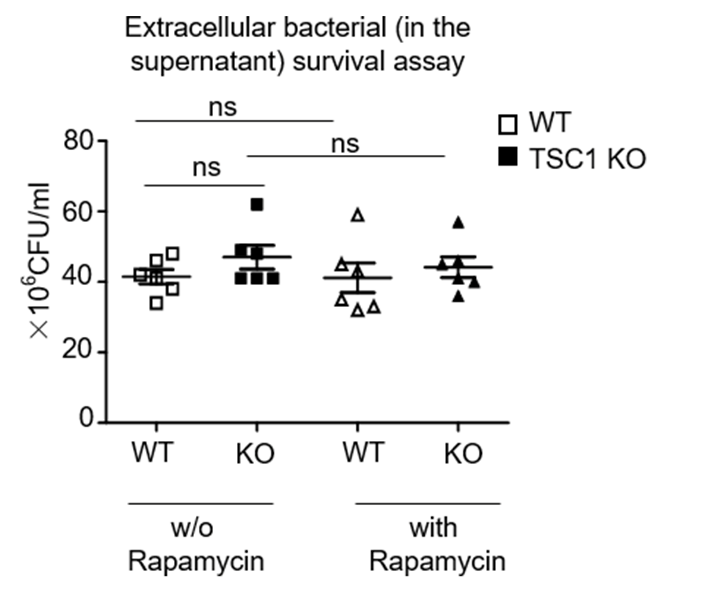


**Supplementary Figure 2.** **The extracellular bacterial counts in the supernatant of V. vulnificus infected WT and TSC1 KO *BMMϕs*.** Extracellular survival assay for viable bacterial counts in the supernatant of *V. vulnificus*-infected WT and TSC1 KO macrophages with or without rapamycin pretreatment. Data shown are representative of at least three experiments. P value was determined by Student *t*-test.


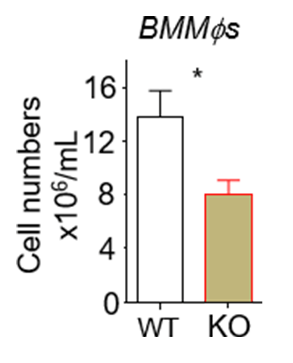


**Supplementary Figure 3.** **The cell number of *BMMϕs* between WT and TSC1 conditional knockout mice.** Data shown are representative of at least three experiments. *, *P* < 0.05 was determined by Student *t*-test.

**2. Supplementary tables**

| **Table 1 Primers used in this study** | |
| --- | --- |
| Primer | Sequence (5’–3’) |
| TNF-α F | CTCCAGGCGGTGCCTATG |
| TNF-α R | GGGCCATAGAACTGATGAGAGG |
| IL-6 F | GCTACCAAACTGGATATAATCAGGA |
| IL-6 R | CCAGGTAGCTATGGTACTCCTGAA |
| iNOS F | CACCAAGCTGAACTTGAGCG |
| iNOS R | CGTGGCTTTGGGCTCCTC |
| IL-1β F | TCACAGCAGCACATCAACAA |
| IL-1β R | TGTCCTCATCCTGGAAGGT |
| Arg-1 F | CCAGAAGAATGGAAGAGTCAGTGT |
| Arg-1 R | GCAGATATGCAGGGAGTCACC |
| CD206 F | TTGGACGGATAGATGGAGGG |
| CD206 R | CCAGGCAGTTGAGGAGGTTC |
| actin F | GGCTGTATTCCCCTCCATCG |
| actin R | CCAGTTGGTAACAATGCCATGT |
